# Submandibular sialolithiasis treatment: a comparative pilot prospective study of holmium: YAG laser and pneumatic lithotripsy techniques

**DOI:** 10.1007/s00405-025-09309-9

**Published:** 2025-03-14

**Authors:** Necdet Özçelik, Husam Vehbi, Elvin Alaskarov

**Affiliations:** 1 Department of Otorhinolaryngology, İstanbul Medipol University Health Care Practice and Research Center Esenler Hospital, İstanbul, Turkey; 2 Department of Radiology, İstanbul Medipol University Health Care Practice and Research Center Esenler Hospital, İstanbul, Turkey

**Keywords:** Submandibular sialolithiasis, Sialendoscopy, Holmium:YAG laser, Pneumatic lithotripsy

## Abstract

**Objective:**

Sialendoscopy is a minimally invasive technique employed to diagnose and treat obstructions in the major salivary glands.Holmium: YAG Laser Lithotripsy (HLL) and Intraductal Pneumatic Lithotripsy (IPL) are increasingly utilized for stone fragmentation; however, comparative studies are still limited. This study aims to prospectively compare the outcomes of HLL and IPL in cases of submandibular sialolithiasis.

**Materials and methods:**

Fifty patients diagnosed with submandibular sialolithiasis were randomly assigned to two groups for treatment with either HLL or IPL. Demographics, stone size and location, operative times, and complications were meticulously documented. Preoperative imaging with ultrasound and computed tomography identified stone parameters. Follow-up assessments occurred at 1 week, 3 months, and 6 months post-treatment, which included a 3-month ultrasound to evaluate for residual stones and assess ductal evaluation.

**Results:**

No significant differences between the groups concerning demographics, stone size, or location (*p* > 0.05). However, the IPL group demonstrated shorter operative times for distal and mid-duct stones (*p* < 0.05). The stone-free rates were 92% in the HLL group and 96% in the IPL group. The HLL group exhibited higher rates of mucosal laceration and ductal perforation, while the IPL group showed a greater potential for stone migration. Postoperative symptom resolution was achieved in both groups. Additionally, quality of life (QOL) scores, assessed using the SF-36 questionnaire, showed significant improvement at 6 months postoperatively in both groups, with no statistically significant differences between them.

**Conclusion:**

Both HLL and IPL are effective treatments for submandibular sialolithiasis. IPL is associated with shorter operative times, especially for distal and mid-duct stones, while the complication rates for both methods remain within acceptable limits.

## Introduction

Submandibular sialolithiasis accounts for approximately 80% of stone-related diseases in the major salivary glands [1]. The implementation of sialendoscopy, whether used alone or in conjunction with surgical techniques, has significantly decreased the rates of gland resection in cases of obstructive sialadenitis. Reports indicate that the gland resection rate for submandibular sialolithiasis, which was 35% prior to the introduction of sialendoscopy, has dropped to 5% with its advent [2].

Organ-preserving approaches, including transoral duct surgery (TDS) performed independently or in conjunction with endoscopic methods, as well as advanced intraductal lithotripsy techniques, are commonly preferred treatment options.Transductal incision is a well-established technique for the removal of submandibular duct stones, particularly when the stones are palpable in the distal portion of Wharton’s duct. This approach involves making a small incision directly over the duct to facilitate stone extraction, often performed under local anesthesia. It is considered a straightforward and cost-effective method, especially for accessible stones, with minimal equipment requirements compared to interventional sialendoscopy [3, 4].

Various types of lasers and pneumatic devices are employed for lithotripsy (stone fragmentation) under sialendoscopic guidance. Numerous studies have demonstrated the individual effectiveness of both holmium laser and pneumatic lithotripsy methods in treating submandibular sialolithiasis [5, 6]. However, a review of the literature indicates that there are only a limited number of studies comparing the efficacy of these two methods.

The aim of this study is to prospectively compare the efficacy of Holmium laser lithotripsy and pneumatic lithotripsy in the treatment of submandibular sialolithiasis, as well as to elucidate the differences between these two techniques.

## Materials and methods

This study was designed as a prospective, single-center pilot study and conducted following approval from the ethics committee (Decision No: E-10840098-772.02-4982) of our clinic. A total of 50 patients scheduled for therapeutic sialendoscopy under general anesthesia due to submandibular sialolithiasis between October 2021 and March 2024 were included in the study. The patients were divided into two groups, receiving treatment with either Holmium: YAG laser or pneumatic lithotripsy. Preoperative data, including age, gender, symptoms, and duration of illness, were recorded for all cases included in the study.

Patients with a history of autoimmune or systemic diseases, insufficient data, or short follow-up periods were excluded from the study. The choice of intervention method was made randomly, and the patients were blinded to the lithotripsy technique that would be employed. Preoperative neck ultrasound and tomography were performed to assess the shape, size, location, and structure of the gland containing the stone. For patients diagnosed with active sialadenitis, the intervention was planned at least one month after the completion of appropriate antibiotic treatment. Postoperative follow-ups were conducted during the first week, as well as at three and six months post-surgery. The presence of residual stones, duct structure, and gland condition were evaluated using neck ultrasound for all patients at the three-month postoperative mark.

**Fig. 1:**
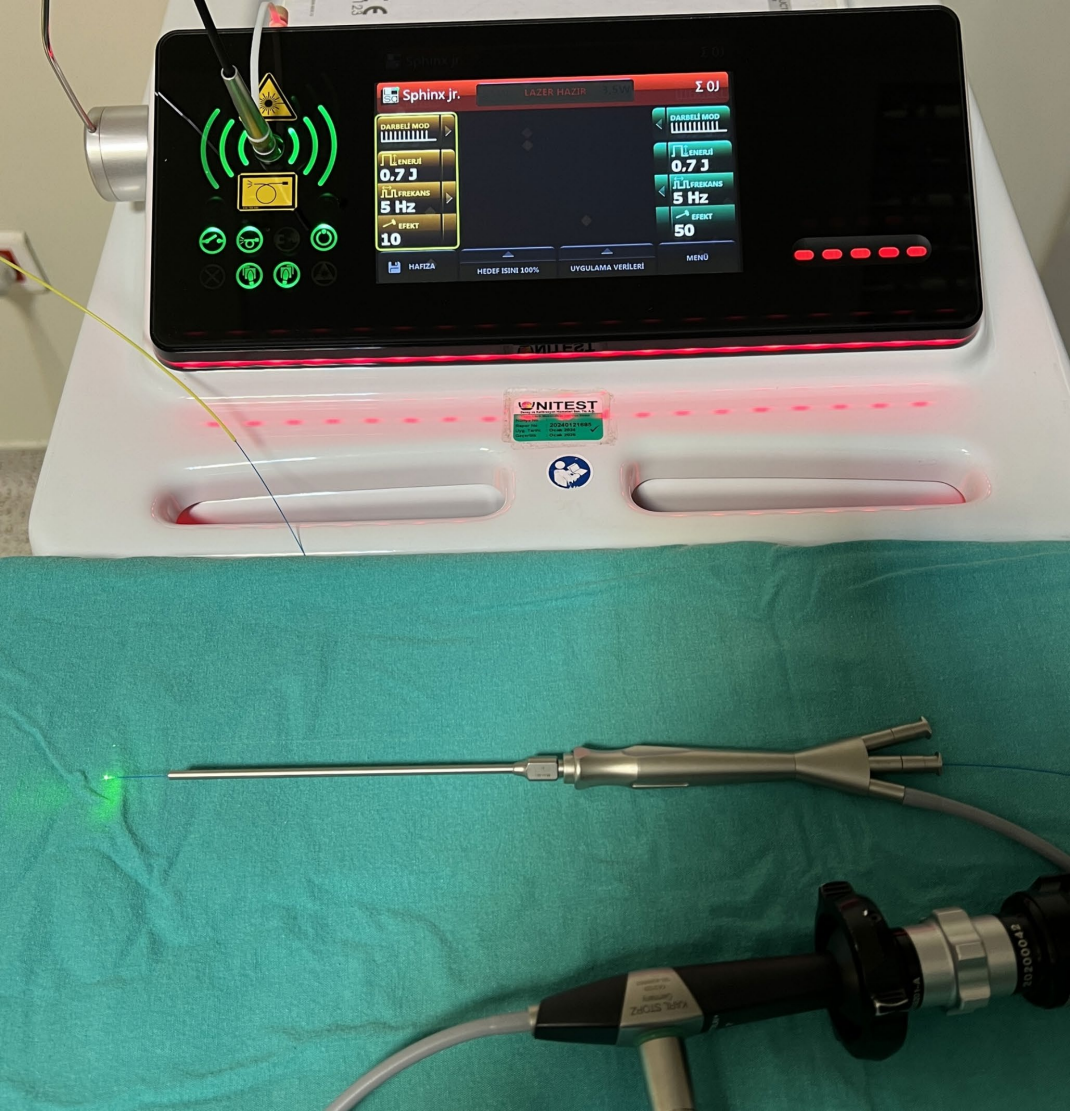
The laser fiber has been passed through the intervention channel of the All-in-one scope and is ready for use

Marshall all-in-one semi-flexible endoscopes with diameters of 1.3 mm and 1.6 mm (Karl Storz, Tuttlingen, Germany) were utilized for sialendoscopy in all patients. For laser lithotripsy, 0.2 mm fiber probes were connected to a Litho (LISA, SPHINX^®^ JR, Germany) holmium laser system. During the procedure, energy levels ranging from 0.5 to 1.5 J were applied at a frequency of 3 to 5 Hz (Fig. 1).

**Fig. 2:**
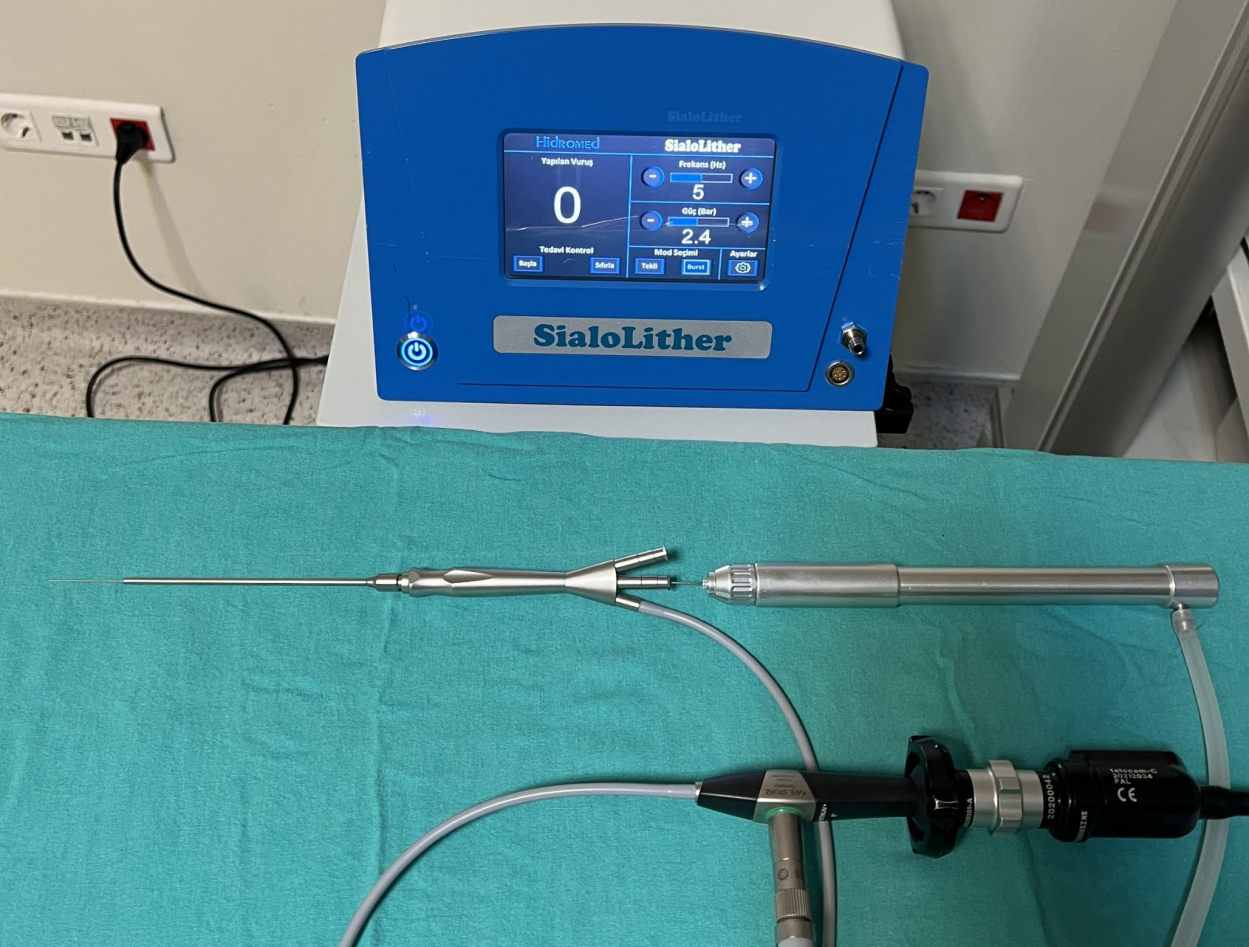
The pneumatic lithotriptor probe is assembled and stone-breaking wire part is passed through the intervention channel of the All-In-One scope and is ready for use

For pneumatic lithotripsy, an electro-pneumatic device (SialoLither, HidroMED, Turkey) with dimensions of 380 × 315 × 215 mm and specially designed probes with diameters of 0.6 mm and 0.7 mm were utilized. During the procedure, a pressure of 3.5 to 4.5 atm was applied for each shot. To remove the fragmented stones, basket catheters measuring 35 cm in length and 0.4–0.6 mm in diameter, or foreign body forceps with a length of 30 cm (Karl Storz), were employed (Fig. 2).

Preoperative, intraoperative, and postoperative data were recorded and compared based on the methods employed. Patients were discharged one day after surgery, and they received amoxicillin-clavulanate and analgesic treatment for one week. During follow-up examinations, we evaluated the complete resolution of preoperative symptoms, the reduction in their frequency, and the presence of any complications.

Postoperative quality of life (QOL) was assessed using the Turkish version of the SF-36 questionnaire, comparing preoperative and 6-month postoperative scores within each group.

Statistical analyses were performed using the independent t-test, chi-square test, Wilcoxon Signed-Rank test, and ANOVA to assess group differences. All results are presented as mean ± standard deviation, and a p-value of less than 0.05 was deemed statistically significant.

## Results

A total of 50 patients diagnosed with submandibular sialolithiasis were prospectively evaluated in two groups: HLL and IPL. No significant differences were observed between the groups concerning demographic and clinical characteristics. Additionally, there were no statistically significant differences in gender distribution, mean age, or patient complaints (pain and swelling) (Table 1; *p* > 0.05).

Among the three patients included in the study, two were diagnosed with active sialadenitis due to subfebrile fever, and one due to severe pain. These patients underwent interventional sialendoscopy one month after receiving appropriate antibiotic and anti-inflammatory treatment.

**Table 1:** Demographic and clinical characteristics of the patients (*n* = 50)

Characteristics	HLL (*n* = 25)	IPL (*n* = 25)	*P* Value
Gender (M/F)	10:15	12:13	0.653
Mean Age	39.56 ± 8.35	37.44 ± 8.64	0.378
Complaints			
Pain and Swelling	19	22	
Swelling Only	6	3	

HLL: Holmium Laser Lithotripsy; IPL: Intraductal Pneumatic Lithotripsy

No significant differences were observed between the two groups regarding stone size, number (single or multiple), location (distal, middle, proximal/hilum), and side (left or right) (Table 2; *p* > 0.05).

**Table 2:** Comparison of stone characteristics

Characteristics	HLL (*n* = 25)	IPL (*n* = 25)	*P* Value
Mean Stone Size (mm ± SD)	8.2 ± 3.5	7.9 ± 4.2	> 0.05
Stone Diameter			
5–7 mm	10	12	> 0.05
7–12 mm	9	8	> 0.05
12–15 mm	5	3	> 0.05
> 15 mm	1	2	> 0.05
Number of Stones			
Single	22	23	> 0.05
Multiple	3	2	> 0.05
Stone Location			
Distal Duct	13	15	> 0.05
Middle Duct	10	9	> 0.05
Proximal/Hilum	2	1	> 0.05
Side of Stone			
Left SMG	14	12	> 0.05
Right SMG	11	13	> 0.05

Holmium: YAG Laser Lithotripsy; IPL: Intraductal Pneumatic Lithotripsy; SMG: Submandibular gland

Surgical durations were compared according to stone localization, and significant differences were observed between the HLL and IPL groups (Table 3).

**Table 3:** Comparison of mean surgical duration by stone localization and size

	Mean Surgical Duration (± SD), min		
Characteristics	HLL (*n* = 25)	IPL (*n* = 25)	P Value
Stone Localization Distal Duct			
Distal Duct	126 ± 18	102 ± 15	0.006^*^
Middle Duct	162 ± 21	132 ± 18	0.008^*^
Proximal/Hilum	174 ± 20	168 ± 22	0.87
Stone Diameter			
5–7 mm	135 ± 25	120 ± 18	0.378
7–12 mm	150 ± 22	145 ± 26	0.586
12–15 mm	178 ± 34	165 ± 27	0.863

SD: Standart Deviation; HLL: Holmium: YAG Laser Lithotripsy; IPL: Intraductal Pneumatic Lithotripsy

^*^ Statistically significant value

**Fig. 3:**
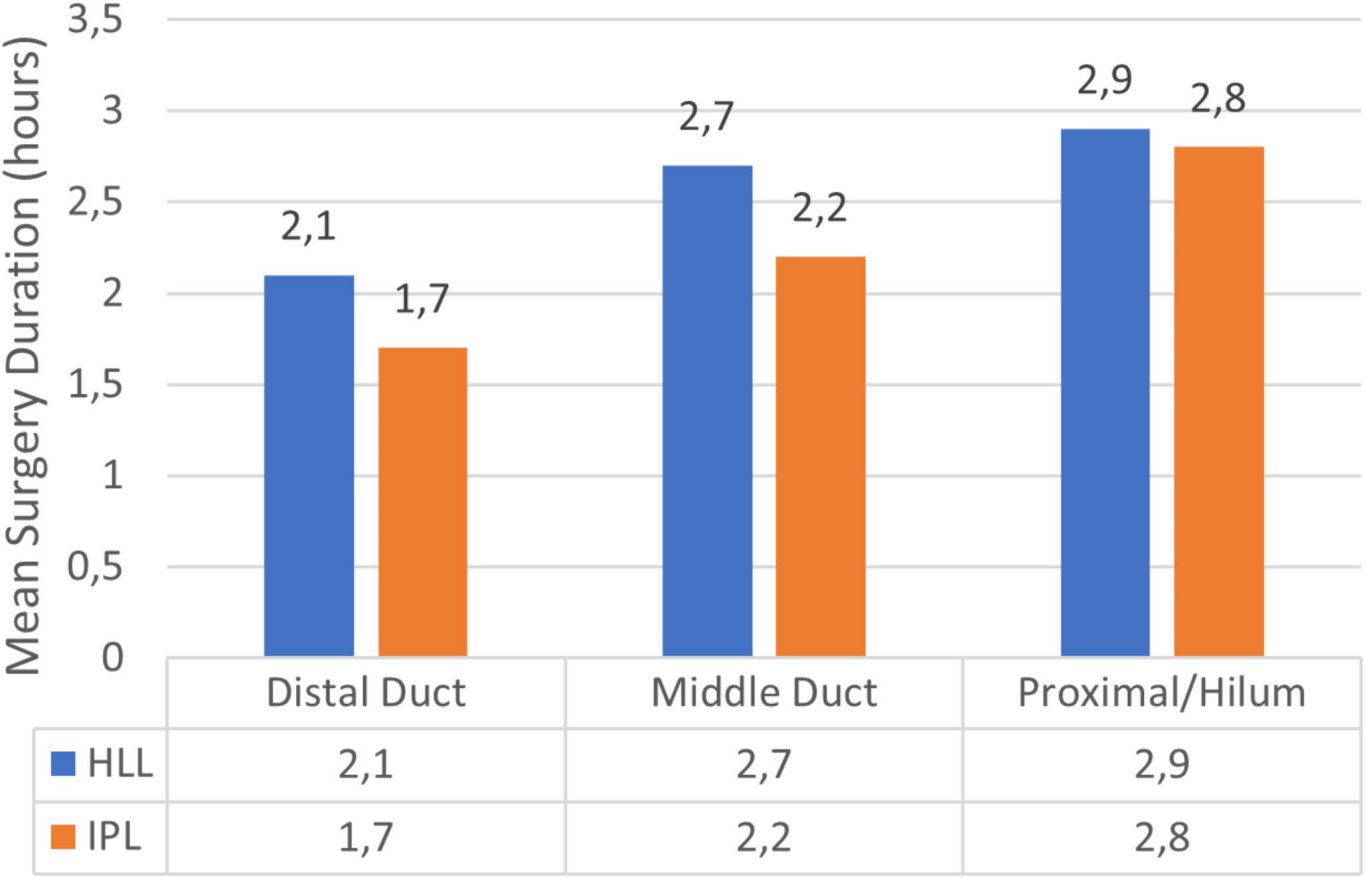
Comparison of Surgical Duration by Stone Localization. HLL: Holmium: YAG Laser Lithotripsy; IPL: Intraductal Pneumatic Lithotripsy

For distal duct stones, the mean surgical duration was 126 ± 18 min in the HLL group and 102 ± 15 min in the IPL group (*p* = 0.006). For middle duct stones, the mean surgical duration was 162 ± 21 min in the HLL group and 132 ± 18 min in the IPL group (*p* = 0.008). However, no significant difference was observed in surgical duration for stones located in the proximal/hilum region (Fig. 3).

The rates of complete stone removal and symptom resolution were high in both groups. In the HLL group, the rate was 92%, while in the IPL group, it was 96% (Table 4).

**Table 4:** Treatment outcomes and complications of submandibular stones

Characteristic	HLL, *n* = 25(%)	IPL, *n* = 25(%)	
Stone free + symptom free	23(92%)	24(96%)	
Symptom free + Residual Stone	1(4%)	1(4%)	
Residual Stone + Persistent Symptoms	1(4%)	0	
Complications			
Mucosal Laceration	2	1	
Ductal Perforation	1	0	
Potential for Stone Migration	1	2	

HLL: Holmium: YAG Laser Lithotripsy; IPL: Intraductal Pneumatic Lithotripsy

All patients were hospitalized for one day postoperatively. Amoxicillin was prescribed for 7 days (to be taken twice daily), along with anti-inflammatory therapy.

Postoperative follow-ups were scheduled for 1 week, 3 months, and 6 months. During these follow-ups, patients were asked about any symptoms resembling the pre-intervention obstruction findings. In the postoperative oral examinations, the glandular duct secretions into the oral cavity were evaluated. This assessment included measuring the quantity of secretion and checking for purulence, which was achieved by massaging the gland or applying lemon juice to stimulate secretion.

When complications were analyzed, two patients in the HLL group experienced mucosal lacerations (without extravasation), and one patient had a ductal perforation in the middle duct region of the left submandibular gland (Table 4). In this case, irrigation was stopped, and a stent was placed proximally to the perforation site. Due to the ductal perforation, the patient experienced dull pain and swelling in the submandibular region beginning on postoperative day one. To reduce gland secretion, one vial (100 units) of botulinum toxin (Botox) was injected under ultrasound guidance, and a tight neck dressing was applied.In the first week following the operation, the symptoms and findings resolved, and the stent was removed. During the one-month follow-up, the patient exhibited normal salivary secretion in the oral cavity, and ultrasound evaluation revealed a normal glandular and ductal structure. Additionally, stone migration to the proximal duct was observed in one patient.

In the IPL group, mucosal laceration was noted in one patient, while stone migration occurred in two patients. In one of these cases, the stone was successfully removed using a basket (Table 4). No serious complications leading to permanent damage were observed in either group.

The SF-36 scores of patients were compared between the preoperative and the 6-month postoperative period. Statistically significant differences were observed in both groups, as presented in Table 5.

**Table 5:** Comparison of preoperative and 6-month postoperative SF-36 scores in IPL and HLL groups

SF-36 Subscale	Preoperative (HLL)	Postoperative (HLL)	*p*-value^*^ (HLL)	Preoperative (IPL)	Postoperative (IPL)	*p*-value^*^ (IPL)
Physical Functioning	60.6 ± 13.1	73.1 ± 10.2	< 0.00001	65.00 ± 12.5	74.3 ± 9.8	0.00026
Role-Physical	31.8 ± 14.2	65.2 ± 11.9	< 0.00001	32.4 ± 15.0	63.7 ± 12.4	< 0.00001
Bodily Pain	44.9 ± 11.5	72.5 ± 7.9	< 0.00001	41.5 ± 10.8	66.9 ± 8.3	< 0.00001

HLL: Holmium: YAG Laser Lithotripsy; IPL: Intraductal Pneumatic Lithotripsy.; SD: Standard Deviation; SF-36:Short form-36

* The Wilcoxon Signed-Rank test was used

Physical functioning scores significantly improved postoperatively in both the IPL and HLL groups (IPL: *p* = 0.00026; HLL: *p* < 0.00001), indicating enhanced mobility and daily activity performance following treatment. A notable improvement was also observed in the Role-Physical subscale, with postoperative scores significantly exceeding preoperative values in both groups (IPL: *p* < 0.00001; HLL: *p* < 0.00001), suggesting substantial postoperative benefits regarding role limitations due to physical health. In the Bodily Pain subscale, both groups demonstrated a highly significant reduction in pain levels postoperatively (IPL: *p* < 0.00001; HLL: *p* < 0.00001), underscoring the efficacy of the procedures in improving patient comfort and reducing pain perception. No statistically significant differences were observed between the HLL and IPL groups in terms of postoperative outcomes.

## Discussion

Sialendoscopy is often the preferred method for diagnosing and treating sialolithiasis. The necessity for lithotripsy during therapeutic sialendoscopy is determined by the size and location of the stone. Small sialoliths (less than 5 mm for the Wharton duct and less than 3 mm for the Stensen duct) can typically be removed easily using instruments such as forceps and baskets [7–9]. We did not require lithotripsy for small stones in our study. Consequently, such cases were excluded from our analysis. Lithotripsy was performed in all cases included in our study.

Thulium and excimer lasers were among the first types of lasers utilized for intraductal lithotripsy. Although positive outcomes have been reported, these techniques have proven to be impractical and costly [10].We have no experience with these specific laser devices, as such lasers were not available in any facility within our university.

Intraductal pneumatic lithotripsy (IPL) and Holmium laser lithotripsy (HLL) are recognized as the most effective and commonly employed intraductal lithotripsy techniques, with both methods demonstrating success rates exceeding 90% [11, 12].

Fragmentation using a Holmium laser is accomplished through the application of photothermal energy. Throughout the procedure, it is essential to maintain good visualization, and the laser energy should be precisely directed at the stone surface to avoid ductal damage. One of the key advantages of laser lithotripsy is the capability of the fine fiber probe to access stones situated in the proximal duct. During the fragmentation process, the “popcorn effect” aids in reducing the stone fragments to significantly smaller sizes, thereby facilitating their easier expulsion from the duct [13, 14].

A study conducted by Giotakis et al. analyzed the prognostic factors influencing the success of sialendoscopy in cases of sialolithiasis, highlighting the significance of stone size, location, and ductal anatomy on procedural outcomes [15]. Our observations indicated that IPL resulted in a shorter operative duration compared to HLL for stones located in the distal and middle ducts, with average surgery times of 102–132 min for IPL and 126–162 min for HLL.

However, Zenk et al. observed a slightly lower IPL success rate of 88% in a larger cohort (*n* = 1,000). They attributed the discrepancies to variations in stone size and operator experience [16]. In our study, no significant difference was observed between the two groups regarding stone size. However, we identified a direct correlation between increasing stone size and prolonged operative duration (Table 3).

Wang et al. investigated the prognostic factors influencing the success of Holmium: YAG laser intraductal lithotripsy, reporting that stone hardness, location, and preoperative imaging findings were crucial to procedural outcomes [17].

Martellucci et al. emphasized the superiority of Holmium: YAG laser lithotripsy for proximal stones, achieving a clearance rate of 89% compared to 76% for IPL [18]. In our series, two patients with proximal/hilar stones were treated using Holmium: YAG laser lithotripsy, while one patient underwent intraductal pneumatic lithotripsy. The mean operative time for these cases was recorded at 2.85 h, with no statistically significant difference observed between the two groups.

In another retrospective study, the outcomes of IPL and HLL were compared in patients with submandibular gland sialolithiasis, showing success rates exceeding 90% for both techniques [19]. In our clinic, Holmium laser lithotripsy has been performed under sialendoscopic guidance since 2014, while the pneumatic device has been utilized since 2018. In our study, we compared the prospective outcomes of both methods, finding that the likelihood of complete stone removal was 92% in the HLL group and 96% in the IPL group.

The integration of IPL or HLL with extracorporeal shock wave lithotripsy (ESWL) or transoral duct surgery (TDS) could enhance outcomes for complex cases. Koch et al. demonstrated a 95% success rate using ESWL followed by IPL for impacted stones, suggesting synergies in multimodal approaches [20].

Similarly, combining HLL with TDS may mitigate challenges in hilar stones, as proposed by Zenk et al. [16].

Future studies should explore hybrid protocols, such as preoperative ESWL for fragmentation followed by endoscopic lithotripsy, to reduce operative times and complications.

Traditional ductal incision, which requires less specialized equipment and time, may appear to be a more cost-effective alternative for stones located in the middle and distal ducts. The average duration of intraductal lithotripsy performed under general anesthesia is approximately two hours, which may be considered a disadvantage when compared to TDS methods. However, in patients with multiple stones, complete removal through ductal incision can be challenging [21]. In our case series, five patients who underwent intraductal lithotripsy had multiple stones. Furthermore, the risk of iatrogenic stenosis following ductal incision should be carefully considered. This type of stenosis may complicate or even impede future sialendoscopic treatments for these patients.

In cases of stones located in the proximal and hilar regions, the use of transoral duct surgery (TDS), either alone or in combination with endoscopy, is often challenging. As a result, intraductal lithotripsy emerges as a more viable treatment option. The success of intraductal lithotripsy in these cases is significantly influenced by the anatomy of the ductal system [21].

Pre-interventional imaging should be employed to assess anatomical variations in the ductal system, including narrow ducts, ductal anomalies, challenging stone locations, or the presence of multiple stones [16]. In our case series, no ductal anomalies were observed in either group. However, multiple stones were identified in five cases.

Intraparenchymal stones are found in 10–20% of sialolithiasis cases. Accessing these stones through interventional sialendoscopy or combined surgical methods is often limited. ESWL is a suitable treatment option for these patients [22]. Notably, no intraparenchymal stones were observed in our study.

Complications associated with sialendoscopy may include sialadenitis, persistent swelling, postoperative stenosis, temporary paresthesia, ductal perforation, ductal ligation, wire basket obstruction, infection, bleeding, traumatic ranula formation, and stone migration [23, 24]. In our study, continuous irrigation with cold saline was performed to prevent thermal damage to the duct walls caused by the laser’s thermal effect. Ductal perforation was observed in one patient, while mucosal laceration occurred in two patients in the HLL group. In contrast, mucosal laceration was observed in one patient in the IPL group.

Strychowsky et al. in a meta-analysis of 1,773 sialendoscopy cases, reported that the overall stone migration rate associated with lithotripsy techniques ranges from 7 to 10%. In comparison to HLL, IPL presents a 1.5-fold increased risk of stone migration [25]. In our case series, stone migration was observed in one patient (4%) in the HLL group and two patients (8%) in the IPL group.

During stone migration, the proactive deployment of basket catheters during fragmentation, as practiced by Marchal et al. effectively captured migrating fragments [26]. In one patient with stone migration, the proximal stone was successfully removed using a basket catheter.

Additionally, temporary balloon occlusion proximal to the stone, as described by Nahlieli et al. may help prevent retrograde migration [27].

When reviewing the urological experience and literature on stone retropulsion, multiple devices and techniques have been developed to prevent the phenomenon before applying kinetic energy to the stone. Some of these include the Lithovac, Passport Balloon, Stone Cone, PercSys Accordion, NTrap, and stone baskets such as LithoCatch, Parachute, and Escape. Additionally, some researchers have explored the use of lubricating jelly and BackStop gel to prevent stone migration. These devices and gels are placed proximal to the stone before energy application to limit its displacement [28, 29].

However, when considering the anatomical constraints of the salivary duct system, we found that our ductal structures are significantly narrower and more rigid compared to the urinary tract. Consequently, incorporating a separate wire or catheter alongside the endoscope within the working channel was deemed impractical, as it would limit maneuverability.

Preventing stone retropulsion during intracorporeal lithotripsy is a well-established concern in urology. However, its adaptation to sialolithiasis remains an area requiring further exploration and research.

Patient satisfaction and postoperative quality of life following sialendoscopy are influenced by several factors, including the type of gland involved (parotid vs. submandibular), the volume of irrigation fluid used, the applied pressure during the procedure, the duration of the intervention, and the number of instrumentations within the duct [30].

In a prospective, non-randomized study conducted by Melo et al., sialendoscopy was reported to significantly improve patients’ quality of life. Their findings highlighted a substantial reduction in symptoms and a notable enhancement in overall quality of life scores among patients treated for obstructive salivary gland disease [31].

In our study, QOL was assessed using the Turkish version of the SF-36 questionnaire preoperatively, at the first postoperative week, and at the sixth postoperative month [32].Although no statistically significant differences were observed between the groups, both showed significant improvements in the Physical Functioning, Role-Physical, and Bodily Pain subscales.

In evaluating the cost-effectiveness of pneumatic lithotripsy (IPL) and Holmium: YAG laser lithotripsy (HLL), several financial factors must be considered. While HLL’s significantly higher initial cost (ranging from $80,000 to $150,000) and annual maintenance expenses (between $5,000 and $10,000) limit its accessibility for some institutions, IPL presents a more budget-friendly profile with a lower initial investment (ranging from $5,000 to $10,000) and minimal annual maintenance costs (between $500 and $1,000). Furthermore, the per-procedure costs are substantially lower for IPL (ranging from $100 to $200) compared to HLL (ranging from $400 to $800). onsidering these factors, IPL may be preferable for high-volume centers prioritizing cost-efficiency, whereas HLL is more advantageous for institutions performing multidisciplinary lithotripsy procedures requiring precise fragmentation.

The present study has several limitations, including its single-center design, a limited patient cohort, and the absence of long-term follow-up (defined as five years or more). While the current investigation evaluated outcomes for up to six months, recurrence rates and late complications (e.g., ductal stenosis) may become apparent later. Literature reports long-term recurrence rates of 5–10% following sialolithiasis treatment [33, 34].

Additionally, future studies with an extended follow-up period of at least 12 months or more are recommended to evaluate recurrence rates and long-term complications. This will provide a more comprehensive understanding of the durability and safety of these techniques in the long term.

Nevertheless, we believe that the homogeneous distribution of patients across groups and the prospective evaluation of results make significant contributions to the existing literature.

In summary, both treatment methods were found to be effective for submandibular sialolithiasis. However, the IPL method offers a shorter surgical duration, particularly for stones located in the distal and middle ducts. The complication rates for both methods remain within acceptable limits, and these findings should be corroborated by larger-scale studies.
